# *In Vivo* Osteogenic and Angiogenic Properties of a 3D-Printed Isosorbide-Based Gyroid Scaffold Manufactured via Digital Light Processing

**DOI:** 10.3390/biomedicines12030609

**Published:** 2024-03-07

**Authors:** Fiona Verisqa, Jeong-Hui Park, Nandin Mandakhbayar, Jae-Ryung Cha, Linh Nguyen, Hae-Won Kim, Jonathan C. Knowles

**Affiliations:** 1 Division of Biomaterials and Tissue Engineering, Eastman Dental Institute, University College London, London NW3 2PF, UK; fiona.verisqa.19@ucl.ac.uk (F.V.); l.nguyen@ucl.ac.uk (L.N.); 2 Institute of Tissue Regeneration Engineering (ITREN), Dankook University, Cheonan 31116, Republic of Korea; shurins@naver.com (J.-H.P.); m.nandia@gmail.com (N.M.); kimhw@dku.edu (H.-W.K.); 3 Department of Biochemistry, School of Biomedicine, Mongolian National University of Medical Sciences, Ulaanbaatar 14210, Mongolia; 4 Department of Chemistry, Dankook University, Cheonan 31116, Republic of Korea; chajaeryung@naver.com; 5 UCL Eastman-Korea Dental Medicine Innovation Centre, Dankook University, Cheonan 31116, Republic of Korea; 6 Department of Nanobiomedical Science & BK21 PLUS NBM Global Research Center for Regenerative Medicine, Dankook University, Cheonan 31116, Republic of Korea

**Keywords:** 3D printing, gyroid structure, bone, implant, *in vivo*

## Abstract

Introduction: Osteogenic and angiogenic properties of synthetic bone grafts play a crucial role in the restoration of bone defects. Angiogenesis is recognised for its support in bone regeneration, particularly in larger defects. The objective of this study is to evaluate the new bone formation and neovascularisation of a 3D-printed isosorbide-based novel CSMA-2 polymer in biomimetic gyroid structures. Methods: The gyroid scaffolds were fabricated by 3D printing CSMA-2 polymers with different hydroxyapatite (HA) filler concentrations using the digital light processing (DLP) method. A small animal subcutaneous model and a rat calvaria critical-size defect model were performed to analyse tissue compatibility, angiogenesis, and new bone formation. Results: The *in vivo* results showed good biocompatibility of the 3D-printed gyroid scaffolds with no visible prolonged inflammatory reaction. Blood vessels were found to infiltrate the pores from day 7 of the implantation. New bone formation was confirmed with positive MT staining and BMP-2 expression, particularly on scaffolds with 10% HA. Bone volume was significantly higher in the CSMA-2 10HA group compared to the sham control group. Discussion and Conclusions: The results of the subcutaneous model demonstrated a favourable tissue response, including angiogenesis and fibrous tissue, indicative of the early wound healing process. The results from the critical-size defect model showcased new bone formation, as confirmed by micro-CT imaging and immunohistochemistry. The combination of CSMA-2 as the 3D printing material and the gyroid as the 3D structure was found to support essential events in bone healing, specifically angiogenesis and osteogenesis.

## 1. Introduction

Autogenous bone grafts have been the gold standard for large bone defect treatment. However, it has been suggested that an autogenous bone graft leads to pain, poor ambulation, and prolonged hospitalisation. To avoid healthy donor site morbidity, 3D-printed patient-specific implants can be an alternative. However, the availability of biocompatible 3D printing materials is still limited.

The triply periodic minimal structure (TPMS) emerges as a cytocompatible computer-aided design (CAD) for 3D printing, including the biomimetic gyroid structure. The gyroid structure has a smooth curvature and concave architecture that can create porous architectures with high porosity and controllable size. These physical properties have been reported to provide a favourable surface for cell attachment, allowing improved cell proliferation [1,2]. The intricate structure of the gyroid design has been successfully 3D printed using a novel isosorbide polymer, CSMA-2. CSMA-2 is an isosorbide-based polymer derived from sustainable sources such as starch and cellulose. Sustainable plant-derived photopolymers offer environmentally friendly materials to substitute fuel-based commercial photopolymers [3]. Isosorbide has desirable stiffness for hard tissue engineering due to its ring structure. In addition, the good optical transparency of isosorbide is also advantageous for its development as a 3D printing material, especially for lithography methods. Isosorbide can serve as the backbone for polymers with methacrylate or acrylate groups as the functional groups [3]. Several studies have demonstrated successful 3D printing with various isosorbide-based composites for hard tissue engineering [3,4,5].

Previous work has also shown that a light-cured CSMA-2 scaffold enables angiogenesis on a chorionic allantoin membrane (CAM) ex vivo assay, supporting the angiogenic potential of the polymer [4]. *In vivo* studies are essential to confirm and evaluate these properties further. Angiogenic properties correlate with the osteogenicity of a bone substitute biomaterial. The osteogenicity of the bone tissue engineering scaffold allows the new bone formation to restore the defect by recruiting stem cells from the surrounding tissue and promoting cell differentiation [6]. This process is also supported by blood vessel formation, particularly in larger defects.

A scaffold’s pore size and interconnectivity have been reported to affect vascularisation and tissue repair [6]. The open architecture of the gyroid surface allows cell suspension infiltration, resulting in a better distribution of cells throughout the scaffolds [7]. The permeability of a gyroid structure also allows a longer cell culture period since the cells seeded on the scaffold can receive adequate nutrients and oxygen. The gyroid structure has a self-supporting feature that eliminates the requirement of building the support structure and reduces material consumption. This feature enables 3D printing with vat photo polymerisation methods such as stereolithography (SLA) and digital light processing (DLP), in which gyroid structures have been successfully printed using photopolymers to be utilised [8,9]. Furthermore, DLP allows 3D printing of high-resolution gyroid structures, which is important to ensure cytocompatible smooth surfaces and curvatures.

Therefore, this study aims to evaluate the *in vivo* osteogenic and angiogenic properties of 3D-printed isosorbide-based CSMA-2 gyroid scaffolds manufactured via the DLP method. 

## 2. Materials and Methods

### 2.1. CSMA-2 Synthesis

CSMA-2 synthesis was conducted following previous methods from Owji et al., 2019 and Shakouri et al., 2020 [10,11]. The first step of the process was the synthesis of BHIS (bis(2-hydroxyethyl) isosorbide) by reacting isosorbide (Sigma Aldrich, Dorset, UK) (100 g, 684.3 mmol) and ethylene carbonate (Sigma Aldrich, Dorset, UK) (132.57 g, 1505.5 mmol) that were degassed under dry nitrogen for 1 h. The reaction was then heated on a hot plate for 1 h at 70 °C. After the components were completely dissolved, the reaction mixture was heated to 170 °C. Then, potassium carbonate (3.0 g, 21.71 mmol) was added, and the mixture was left to react for 48 h. The resulting BHIS was purified through silica column chromatography using methanol and ethyl acetate (1:9). The purified BHIS was then evaporated in a rotary evaporator to remove the solvents. 

The next step was reacting the purified BHIS (32.15 g, 79.37 mmol) with IPDI (Sigma Aldrich, Dorset, UK) (57.15 g, 257.07 mmol), TEGDMA (Sigma Aldrich, Dorset, UK) (125 g, 436.56 mmol), and five drops (approximately 0.5 mL) of dibutyl tin dilaurate (DBTDL) Sigma Aldrich, Dorset, UK) at 25 °C for four hours. After that, HEMA (Sigma Aldrich, Dorset, UK) (71.42 g, 548.82 mmol) and another 5 drops (approximately 0.5 mL) of DBTDL were added into the reaction mixture and left to react for 12 h at 25 °C, resulting in the final CSMA-2 monomer ((3R,3aR,6S, 6aR)-hexahydrofuro [3,2-b] furan-3,6-diyl)bis(oxy)) bis(ethane-2,1-diyl))bis(oxy))bis(carbonyl))bis(azan ediyl))bis(3,3,5-trimethylcyclohexane-5,1-diyl))bis (azanediyl))bis(carbonyl))bis(oxy))bis(ethane-2, 1-diyl) bis(2-methylacrylate)).

Phenylbis(2,4,6-trimethyl benzoyl)phosphine oxide or BAPO (Sigma Aldrich, Dorset, UK) was used as the photoinitiator to develop photo curability. For this experiment, 2 wt% of BAPO was added to the CSMA-2 and left to stir for 24 h. Hydroxyapatite or HA (Captal R, Plasma Biotal, Derbyshire, UK) with a 1.67 Ca:P ratio and particle size ranging from 6 to 20 μm was added and mixed into the CSMA-2 using a speed mixer at 1700 RPM for 2 min. HA addition to the CSMA-2 was 10% *w*/*w*. The final CSMA-2 groups were CSMA-2 0HA (without HA) and CSMA-2 10HA (10% HA). The HA percentage was determined based on a previous study [5].

### 2.2. Scaffold Fabrication

Gyroid bone scaffolds were 3D printed using CSMA-2 and a Nobel Superfine DLP 3D printer (XYZ Printing, New Taipei City, Taiwan). The scaffolds were fabricated with and without adding 10% *w*/*w* hydroxyapatite (HA). For the subcutaneous model, the scaffolds were 6 mm in diameter and 6 mm in height. To observe bone remodelling, scaffolds with a 5 mm diameter and 2 mm size were 3D printed.

A slicing software (XYZware Nobel 1.1.44.9, XYZ Printing, New Taipei City, Taiwan) was used to slice the design made by computer-aided design (CAD) software (Meshmixer 3.5, Autodesk, San Francisco, CA, USA) and determine the printing setup. The base setup’s curing time was 19 s with a power intensity of 60 W·m^−2^. The curing time for intermediate and model setups was 8.3 s with a power intensity of 53 W·m^−2^. All setups used 15% of the power level and a 0.25 mm·s^−1^ speed at 20 °C. The printing resolution or layer height was set at 50 μm.

After printing, the samples were washed with 99% methanol (Merck, Darmstadt, Germany) for 5–10 min to remove uncured monomers. Subsequently, they were left to dry and underwent a post-curing process with a UV chamber (XYZ Printing, New Taipei City, Taiwan) for 1 min at level 3 UV LED power intensity (16 watts). Before implantation, the samples were sterilised using ethylene oxide (EO) gas.

### 2.3. Scaffold Characterisation

#### 2.3.1. Surface Morphology

Scanning electron microscopy (SEM), using the Zeiss Sigma 300VP (Carl Zeiss Ltd., Cambourne, UK), was used to evaluate the scaffold morphology. Before the analysis, samples were coated with 95% gold and 5% palladium (Polaron E5000 Sputter Coater, Quorum Technologies, Laughton, UK). The printing resolution was observed by measuring the layer thickness.

#### 2.3.2. Wettability

The wettability of the 3D printed scaffold was examined by calculating the surface energy of the 3D-printed flat sample surface. Ultrapure water (NANOPure Diamond, Barnstead, NH, USA), glycerol (Sigma-Aldrich, St. Louis, MO, USA), and di-iodomethane (GPR) contact angles were obtained using KSV instruments’ Cam 200 optical contact angle meter (Biolin Scientific, Manchester, UK). The angles were obtained upon contact of the flat specimen surface with the liquid droplet and measured between the specimen’s surface–liquid interface and the liquid–air interface.

#### 2.3.3. Mechanical Properties

A compressive test was performed using Shimadzu Autograph AGS-X machinery (Shimadzu, Milton Keynes, UK). Preload was performed at 3 mm·min^−1^ speed with a maximum force of 1 N. Next, the cylinders were compressed with a 2 kN load cell and parallel loading plates at a 1 mm·min^−1^ crosshead speed until the sample failed. Gyroid cylinders with six replicates were used as samples. The data were obtained via Trapezium Lite X v1.3.1 software (Shimadzu, Milton Keynes, UK).

### 2.4. Scaffold Implantation

Small animal models used in this study were 6-week-old (subcutaneous model) and 10-week-old (calvaria model) male Sprague-Dawley (SD) rats. A 20–24 °C fixed temperature with 30–70% humidity was maintained. The nutrients comprised standard pellet foods and water. Light and dark cycles, 12 h each, were also provided.

Before the surgical procedure, the rats were anaesthetised with 2.5% isoflurane (Forane, Choongwae Pharma, Gwacheon-si, Republic of Korea) in a 2:1 mixture of nitrous oxide to oxygen. The hair was shaved, and the surgical area was wiped with iodine and 70% ethanol. The skin incision was performed using a scalpel.

To perform the subcutaneous tissue compatibility model implantation, 20 mm incisions were made to each dorsal side of the rats. Six sites for subcutaneous pockets were created using Metzenbaum scissors and blunt dissection on the back, laterally from the spine of each rat.

A rat calvaria critical-size defect model was used for the bone regeneration analysis. Two 5 mm calvaria defects positioned on either side of the parietal bone were produced in each rat using a dental handpiece and a 5 mm trephine bur under cooling conditions with sterile saline. Before implantation, the defect sites were randomly allocated to experimental groups (Sham, CSMA-2 0HA, and CSMA-2 10HA).

After the implantation, the skin was sutured with non-absorbable sutures (4-0 Prolene, Ethicon, Norderstedt, Germany), and the subcutaneous model was sacrificed at 1, 7, 14, or 21 days, while the calvaria model was sacrificed after 6 weeks by carbon dioxide inhalation.

### 2.5. Scaffold Evaluation

The implant and surrounding tissue were extracted and fixed in 10% neutral buffered formalin for 24 h at room temperature.

The subcutaneous models were soaked in 10–30% sucrose solution for one day and stored at −80 °C in an optimal cutting temperature compound (Tissue-Tek, Sakura, Torrance, CA, USA). The frozen blocks were sectioned into 8 μm slices using a cryostat microtome (Leica, Wetzlar, Germany). The sliced samples were mounted on slides and stained with haematoxylin and eosin (H&E) to analyse tissue fibrosis and neovascularization.

New bone formation on the calvaria model was first visualised with a micro-CT scanner (Skyscan 1176, Skyscan, Antwerp, Belgium). The bone formation was measured in a cylindrical region of interest based on the 3D images reconstructed using the Skyscan program. Bone volume (BV), bone surface (BS), bone volume/tissue volume (BV/TV), and bone surface density (BSD) were analysed quantitatively.

The samples were decalcified with rapid Cal solution for histology and immunohistochemistry analyses. The decalcified samples were dehydrated using ethanol and xylene and embedded in a paraffin solution. The paraffin blocks were sectioned into 5 μm slices using a semi-automated rotary microtome (RM2245, Leica, Wetzlar, Germany). The sliced samples were mounted on slides and stained with H&E, Masson’s trichrome (MT), and bone morphogenetic protein (BMP) (Invitrogen, MA, USA) to analyse osteogenesis and neovascularization. Images were obtained using a slide scanner (VS200, Olympus, Tokyo, Japan) and analysed using an Olympus image viewer (Olyvia, Olympus, Tokyo, Japan). A designated region of interest on five microscope fields of each specimen was measured for quantification analysis.

### 2.6. Statistical Analysis

The data are presented in the form of mean ± standard deviation. The results were statistically analysed using one-way analysis of variance (ANOVA) at a 95% confidence interval with Tukey’s post hoc test. GraphPad Prism 10 was used to perform the statistical analysis.

### 2.7. Ethics Statement

The animal study was reviewed and approved by the Dankook University Ethics Committee (DKU-18-032). This study was conducted following ARRIVE guidelines.

## 3. Results

### 3.1. 3D Printed Gyroid Scaffold

**Figure 1:**
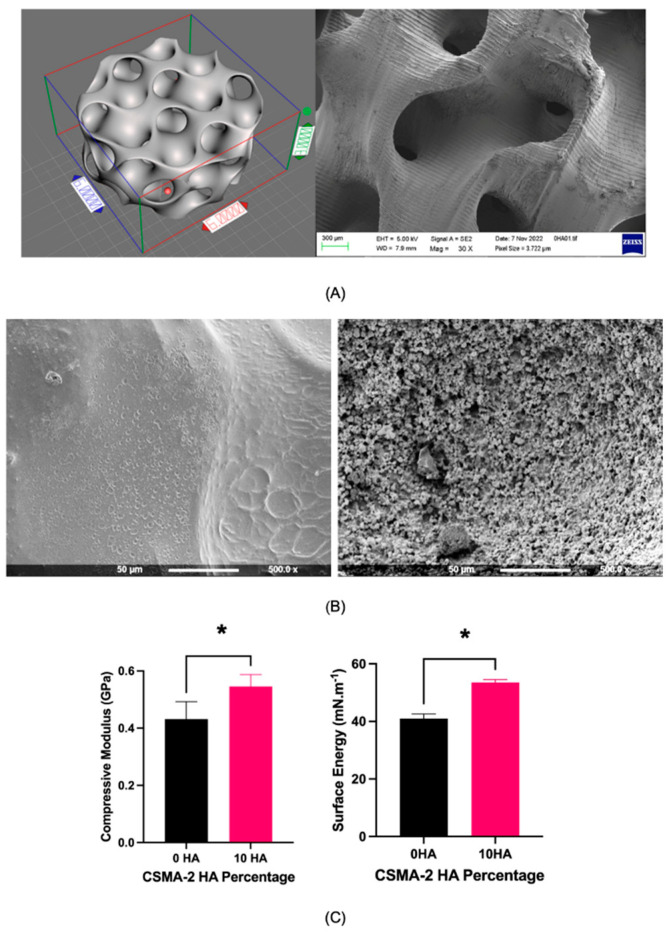
(**A**) The computer aided design (top left) and SEM images of the 3D-printed gyroid scaffold (top right). Gridlines are 1 mm, and the scale bar is 300 μm. Reproduced from Verisqa et al., 2022 [5]. (**B**) Surface morphology of the 3D printed gyroid scaffold obtained via SEM (left CSMA-2 0HA, right CSMA-2 10HA). The surface roughness was visibly different. (**C**) Compressive modulus and the surface energy of 3D-printed CSMA-2 gyroid scaffold. Data are presented as mean ± standard deviation. * = *p* < 0.05.

A complex gyroid structure with interconnected pores was successfully printed using DLP and CSMA-2 as the photopolymer. Figure 1A illustrates CAD and 3D printed scaffolds, highlighting apparent similarities in dimension and architecture between the design and the printed construct. The surface morphology of the scaffold groups was visibly different, with CSMA-2 10HA showing a rougher and irregular surface, as can be seen in Figure 1B. CSMA-2 10HA also demonstrated a higher compressive modulus and surface energy.

### 3.2. Angiogenesis

**Figure 2:**
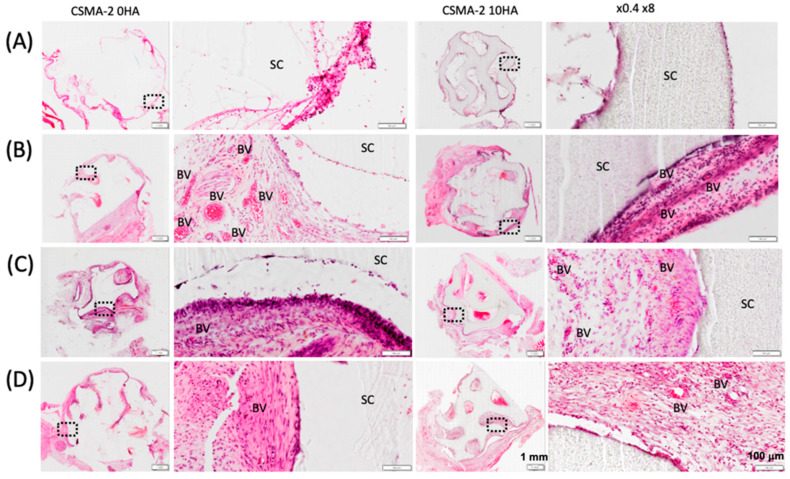
Angiogenesis on CSMA-2 0HA and 10HA scaffolds implanted in rat subcutaneous model for (**A**) 1 day, (**B**) 7 days, (**C**) 14 days, and (**D**) 21 days. H&E stain showing histological low and high magnification images of the interface between connective tissue, blood vessels (BV), and scaffold (SC). Scale bars are 200 μm in high magnification and 1 mm in low magnification.

On day 1 post-implantation, CSMA-2 0HA and 10HA samples did not show visible angiogenesis (Figure 2A). The blood vessels were visible on day 7 samples, as shown in Figure 2B. The neovascularisation began from the scaffolds’ peripheral area on day 7 and progressed into the internal porous structure on day 14 and 21, as Figure 2C,B reveal. The capillary blood vessels with various diameters were found to infiltrate the gyroid structure of CSMA-2 10HA scaffolds more than CSMA-2 0HA group. Both scaffold groups demonstrated close contact of the implanted scaffolds with the surrounding connective tissue and newly formed blood vessels.

**Figure 3:**
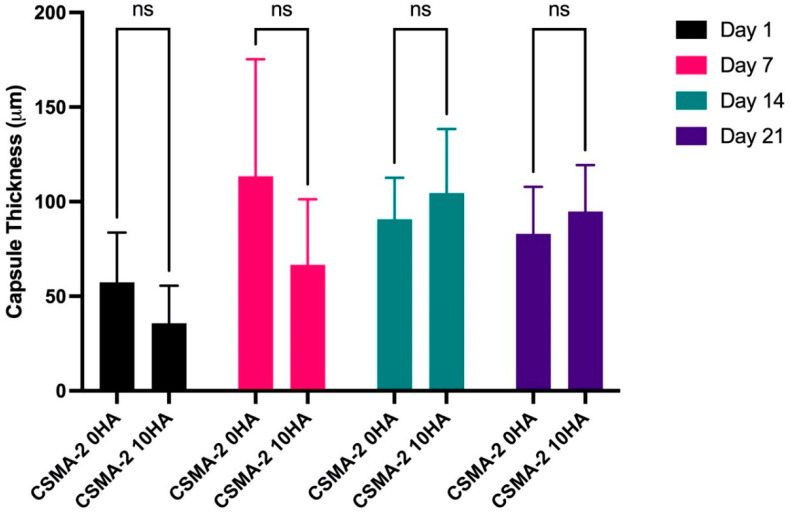
Quantification of the thickness of the fibrous capsule. Data are presented as mean ± standard deviation. ns = not significant.

Fibrous capsule formation was evaluated to analyse the tissue response to the scaffolds after the surgical implantation. CSMA-2 0HA scaffolds generated visually thicker fibrotic capsules at earlier time points, days 1 and 7 (Figure 3). For the CSMA-2 10HA group, day 14 samples had the thickest fibrous capsule, which decreased on day 21. Quantitatively, the fibrosis thickness did not differ significantly among the groups in the following days.

### 3.3. New Bone Formation

**Figure 4:**
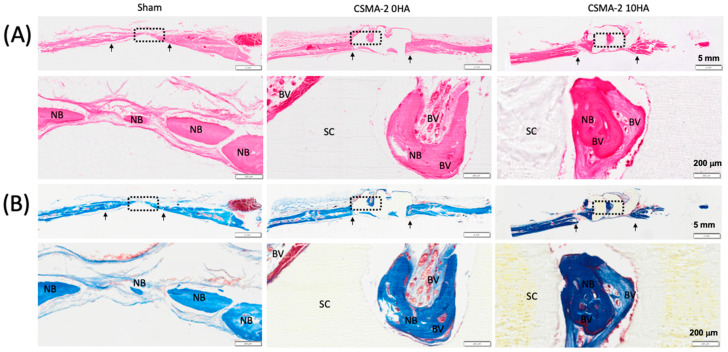
(**A**) H&E stain and (**B**) MT stain at low and high magnification. New bone (NB), scaffold (SC), blood vessels (BV), and black arrows indicate the margins of the bone defect site. Scale bars are 200 μm in high magnification and 1 mm in low magnification.

A histological analysis was performed to evaluate new bone formation on the rat calvaria critical-size defect 6 weeks after surgical implantation. New bone tissue was visible in every surgical group, with sham surgery as the control group showing relatively thinner tissue compared to the defect with scaffold implantation (Figure 4A). Newly formed blood vessels were also seen to infiltrate CSMA-2 0HA and 10 HA samples, although they were not noticeable in the sham surgery group. Masson’s trichrome (MT) staining images confirmed the new bone formation, with the positive blue stain indicating the abundant new collagen fibres (Figure 4B).

**Figure 5:**
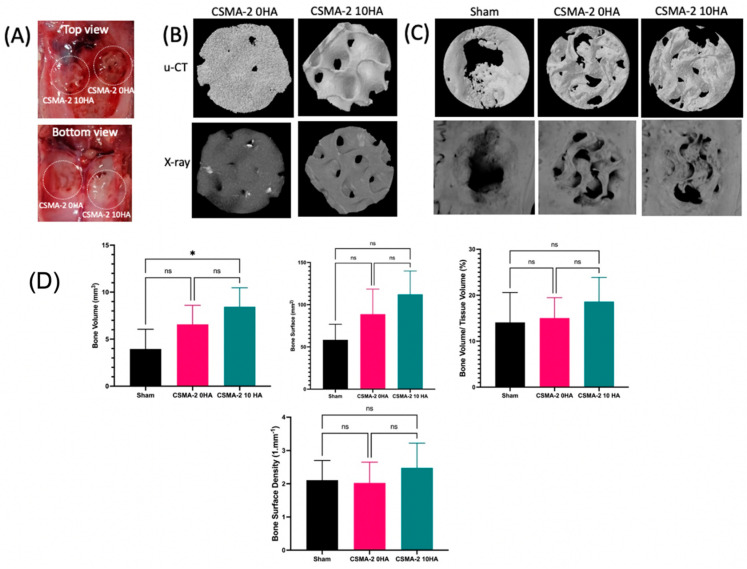
The osteogenic capacity of the CSMA. CSMA-HA implanted in rat calvaria defect model for six weeks. (**A**) Optical images, (**B**) 3D scaffold μ-CT images, (**C**) μ-CT images were taken to reveal new bone, (**D**) μ-CT quantitative analyses of bone volume (BV), bone surface (BS), bone volume/tissue volume (BV/TV), and bone surface density (BSD). Data are presented as mean ± standard deviation. ns = not significant. * = *p* < 0.05.

Optical images (Figure 5A) reveal new bone formation at the implant site, which is further corroborated by the X-ray and micro-CT images (Figure 5B,C). The quantified volume of new bone formation was higher in the CSMA-2 10HA group than in CSMA-2 0HA, with volumes of approximately 8 mm^3^ and 6 mm^3^, respectively. Both scaffold groups exhibited a greater quantified bone surface than the sham surgery group, measuring roughly 80 mm^2^ for CSMA-2 0HA and 110 mm^2^ for CSMA-2 10HA (Figure 5D). CSMA-2 10HA samples also demonstrated the highest bone volume fraction (BV/TV) and bone surface density (Figure 5D), whilst CSMA-2 0HA showed the lowest value of those new bone formation parameters among the surgical groups. The bone volume fraction of CSMA-2 10HA was around 18%, which did not differ significantly from the sham surgery control group.

**Figure 6:**
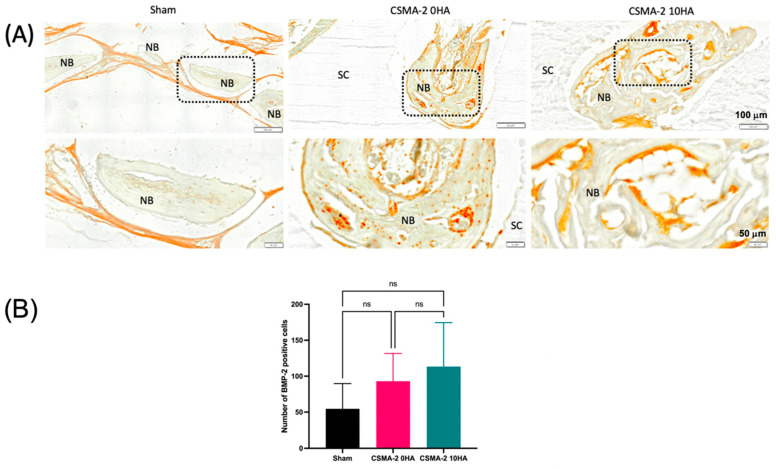
Immunohistochemical expression of BMP-2 protein at the newly formed bone areas in the calvaria defect (**A**). Quantification of BMP-2 expression from the cells (**B**). Scale bars are 200 μm in high magnification and 1 mm in low magnification. Data are presented as mean ± standard deviation. ns = not significant.

New bone tissue formation was also evaluated using immunohistochemical bone morphogenetic protein-2 (BMP-2) staining. Positive BMP-2 staining is evident from the brownish colour on the images (Figure 6A). The sham surgery control group demonstrated weaker positive BMP-2 staining than the scaffold groups, with sporadic positive staining observed in CSMA-2 0HA group. Positive staining of BMP-2 was also observed on the porous structure of CSMA-2 10HA scaffolds. A quantitative analysis of BMP-2 staining revealed that the highest number of positive cells are on the CSMA-2 10HA scaffold, followed by the CSMA-2 0HA scaffold and the sham surgery group (Figure 6B).

## 4. Discussion

As depicted in Figure 2A, the connective tissue became visible from day 1 post-implantation. The tissue effectively infiltrated the porous architecture of the 3D-printed gyroid scaffold, indicating the importance of appropriate pore size in inducing the necessary tissue response in the initial anabolic phase of bone healing. The increase in fibrotic tissue volume can also be observed following the implantation of the 3D-printed gyroid scaffolds in the subcutaneous model (Figure 3). The early stage of bone healing typically involves the formation of a fibrosis capsule, with the soft callus and the subsequent fibrous tissue development occurring around day 5–10 and day 10–16 of the healing process, respectively [12,13]. In this study, the fibrotic capsule was the thickest at day 7 for the CSMA-2 0HA scaffolds and day 14 for the CSMA-2 10HA group. These findings align with the expected timeline for normal wound healing.

Fibrous connective tissue is a hallmark of the fibrovascular phase, a stage characterised by vascular remodelling [14]. Neovascularisation, evident in Figure 2B on day 7 after the surgical implantation, signals the initiation of the fibrovascular phase. This phase encompasses both angiogenesis and vasculogenesis [14]. Angiogenesis forms new blood vessels by sprouting them from the existing vasculature, whilst blood vessels in vasculogenesis are formed by in situ endothelial progenitor cells (EPC) [14]. Angiogenesis involves endothelial cell sprouting, branching, lumen formation, and remodelling [15]. As revascularisation progresses into the injured area and the collagen matrix is deposited, the fibrovascular phase is succeeded by the repair phase, lasting for a few weeks [16]. Blood vessels are still present in the repair phase due to the essential role of vascularisation in facilitating new bone formation. The interdependence of angiogenesis and osteogenesis has been documented in previous studies [17,18]. Disruption of revascularisation can impede the progression of bone healing, potentially leading to non-union fractures or osteonecrosis.

This relationship also applies to hard tissue engineering. One of the significant clinical challenges of bone graft implantation is maintaining the cell viability in the internal part of the graft, which depends on the vascularisation from the recipient site [17]. The lack of vascularisation inside the graft can lead to poor oxygen supply and nutrient transport, and cell death. Further blood vessel infiltration can be seen in Figure 1C. The gyroid structure with approximately 400 μm pore diameter was found to be favourable for vascular remodelling, as shown by the evident blood vessels in the internal structure of the 3D-printed scaffolds.

Corroborating these results, several studies have reported the angiogenic properties of the gyroid structure, noting that pore size and interconnectivity have essential roles in blood vessels [19,20,21]. Scaffolds with a 200–400 μm pore diameter demonstrated the formation of a large blood vascular network of vessels with deep penetration depth [20]. In addition, the geometry of the porous structure has been reported to influence neovascularisation. Scaffolds with hexagonal-shaped pores demonstrated angiogenesis at a significantly slower rate than the scaffolds with gyroid-shaped pores [20]. This finding is consistent with more recent studies, which showed enhanced HUVEC migration and tube formation in gyroid scaffolds compared to cubic and cylindrical pore scaffolds [21].

Several factors could cause these results; one of them is the influence of biomaterials on the Hippo pathway YAP (yes-associated protein)/TAZ (transcriptional coactivator with PDZ-binding motif) [15]. The YAP/TAZ pathway has been reported to regulate endothelial cell proliferation, migration, and viability [15]. YAP/TAZ directly binds to the TEAD family and RUNX family of transcription factors. The interaction of YAP/TAZ with TEAD1 increases the expression of the ANKRD1 gene, leading to the proliferation and angiogenesis of stem cells [22]. Furthermore, osteogenesis is also increased by the binding of YAP/TAZ to the RUNX2 [22]. Therefore, angiogenesis and osteogenesis can be mediated by the YAP/TAZ pathway.

YAP/TAZ functions as a mechanotransducer, influencing cellular responses to the physical microenvironment [23]. YAP and TAZ use the physical cues on the cells and translate them into transcriptional responses [24]. Physical structures, such as the pore curvature of the gyroid design, were identified as activators of the YAP/TAZ pathway [21]. This finding aligns with another study that showcased the activation of the YAP/TAZ pathway by the shallow curvature of the scaffolds’ micropore with a larger diameter (>200 μm) [25]. Moreover, larger pores were associated with robust vascularisation in the subcutaneous animal model [26]. The blood vessel infiltration in larger micropores was deeper than in smaller diameters, and the diameter of blood vessels was also greater in larger pores [26]. Thus, these results indicated positive modulation of cell and tissue responses by the scaffold’s pore size and geometry.

The favourable gyroid architecture was accurately 3D printed using the isosorbide-based polymer CSMA-2. Isosorbide is used as a cardiovascular medicine in isosorbide dinitrate (ISDN) or isosorbide mononitrate (ISMN) form. Previous studies revealed that ISMN could promote angiogenesis in vitro and in zebrafish embryo models, suggesting its potential to treat angiogenesis-related diseases [27]. Isosorbide was also incorporated into a composite scaffold for vascular regeneration. The combination of PLGA and poly(isosorbide sebacate) (PISEB) was shown to be cytocompatible with HUVEC and showed pro-angiogenic gene expression [28]. Moreover, CSMA-2 demonstrated an angiogenic response that can be observed via CAM assay and IHC analysis of CD-31 markers, with or without calcium phosphate addition [4].

In addition, bioceramics such as beta-tricalcium phosphate (β-TCP) have exhibited angiogenic properties by promoting angiogenesis in HUVEC. β-TCP was found to interact with the PI3K/Akt/eNOS axis, promoting vascularisation [29]. Other studies incorporating HA as the bioceramic component demonstrated compatibility with HUVEC, an increased expression of VEGF, and the formation of tubular networks [30]. These in vitro findings suggested the angiogenic properties of HA. Similarly, results from the CAM assay following the implantation of a heparinised chitosan/hydroxyapatite scaffold implantation showed vascular formation, irrespective of the amount of loaded heparin [31]. This outcome indicated the role of HA in angiogenesis by interacting with molecules from microvascular cells and modulating their behaviour [31]. Therefore, combining the 3D printing material and architecture effectively promoted the necessary vascularisation for bone regeneration.

The 3D-printed CSMA-2 gyroid scaffolds also promoted bone growth in critical-size defects, as depicted in Figure 5A–C. Compared to the sham surgery group, the CSMA-2 10HA scaffold group exhibited significantly higher levels of new bone formation. This result corroborated the findings in the previous research, where there was a significant difference in BV/TV between the gyroid scaffold, commercial graft, and the sham after 6 weeks of implantation [32]. The gyroid structure provides a more concave structure, which is more favourable for tissue formation than the convex structure [32]. The smooth surface transition in the gyroid also allows better cell spreading.

Furthermore, cell spreading has been reported to increase YAP/TAZ accumulation in the cell nucleus and activate their transcription [33]. YAP/TAZ is a co-regulator of transcription factors essential for bone homeostasis [22]. They promote stem cell expansion and control osteogenic or chondrogenic differentiation in response to biomechanical and biochemical stimulation [24]. The 3D-printed CSMA-2 gyroid scaffolds provided a favourable physical environment to activate the YAP/TAZ pathway with their stiffness and curvatures. The interaction between cells and the scaffold then leads to the upregulation of YAP/TAZ and their binding to RUNX2, resulting in increased bone growth.

Compared to scaffolds without HA, those with HA showed higher new bone formation, as shown in Figure 5D. HA is a naturally occurring and the most stable form of calcium phosphate, constituting most of the human bone’s inorganic component [34]. It can chemically bond to the bone, demonstrating low toxicity and inflammatory response while influencing osteoblast behaviour to stimulate bone growth [35]. Its composition of calcium and phosphate ions ensures no adverse reactions upon implantation in the human body [36]. HA-based bone graft implantation has been reported to demonstrate bone formation in various animal models [35,37]. New bone formation was observed in the canine alveolar socket as early as 4 weeks post-implantation of different HA forms [35]. Bone regeneration also occurred in rat calvaria critical-size defects implanted with commercial HA products [37,38].

It has been known that HA coating can improve implant integration with bone [36]. After the implantation, the HA surface is covered rapidly by proteins and other biomolecules, leading to the precipitation of calcium phosphate crystal growth or crystal morphology alterations [39]. The surface roughness resulting from HA crystals provided a suitable environment for osteogenic differentiation in a YAP/TAZ-dependent manner [23]. The surface roughness shown by 3D-printed CSMA-2 10HA scaffolds and new bone formation results aligned with these studies. Progenitor cells, such as stem cells, can sense the topography through contact with the material surface, forming lamellipodia and filopodia [40,41]. The topographical information allows filament maturation and forms focal adhesion that induces a specific cellular function [41]. The focal adhesion properties are usually related to actin contractility and Ras homolog family member A (RhoA). RhoA and Rho-associated kinase (ROCK) pathway activation leads to cell spreading. As mentioned before, cell spreading promotes osteogenic differentiation. Focal adhesion kinases (FAK) can also activate the Ras pathway that stimulates the ERK signalling cascade, resulting in the expression of osteogenic markers such as COL I and OCN [42].

The interaction between cells and biomaterials is based on the complex biomolecular interplay between the surfaces [43]. The biological influence of a biomaterial on cells arises from functional protein adsorption and conformational changes of the adsorbed target protein caused by the biomaterial properties. One of the pivotal proteins in bone regeneration is bone morphogenetic protein-2 (BMP-2). BMP-2 is a transforming growth factor β (TGF-β) superfamily member. It plays a crucial role in the formation, growth, development, and reconstruction of bone and cartilage. Additionally, it can stimulate non-osteogenic cells to differentiate into osteoblasts. BMP-2 is essential for organ development in the embryonic stage, such as digit formation and cardiogenesis [44]. In adults, various cells, including osteocytes and osteoblasts, express BMP, contributing to the ossification process. BMP-2 binds with the SER-THR receptor, initiating the osteoblast differentiation signal cascade. The binding leads to the recruitment and phosphorylation of the Smad family, particularly Smad1/5/8 [45].

It has been reported that BMP-2-induced osteogenic differentiation is dependent on cell shape, cytoskeletal tension, matrix stiffness, and cell–ligand interactions [45]. These factors also affect the previously mentioned YAP/TAZ pathway. YAP/TAZ is known to read the cytoskeletal tension, which induces the YAP/TAZ nuclear localisation and crosstalk with the BMP-2 signalling pathway [45]. Previous studies revealed that the knockdown of YAP/TAZ resulted in ALP activity, whilst the recovery of YAP/TAZ increased the ALP [45]. Thus, tension-activated YAP/TAZ was found to regulate BMP-2 signalling and osteogenic differentiation.

Figure 6 reveals the influence of 3D-printed CSMA-2 on BMP-2-induced bone regeneration. Without incorporating exogenous BMP-2, such as FDA-approved RhBMP2, all groups showed positive BMP-2 expression. Moreover, the CSMA-2 10HA group showed the highest BMP-2 positive cells. This result was in accordance with the highest bone volume and fibrous capsule thickness found in the CSMA-2 10HA group. BMPs have been documented to have a role in bone regeneration by recruiting mesenchymal cells and differentiating them into bone cells [46]. BMPs also contribute to bone matrix production and vascularisation [46].

Furthermore, BMP-2 was found to regulate the expression of other BMPs [47]. In a mouse fracture healing study, BMP-2 was found to initiate the repair cascade, showing the peak of its mRNA expression 24 h after the injury [47]. In a human study, BMP-2 expression increased in the endochondral ossification area [47]. These findings explain the expression of endogenous BMP-2 in all groups after 6 weeks of surgery, including the sham surgery group.

It has been documented that there is a strong affinity between HA and the BMP-2 protein [48]. Previous studies investigated the interaction between the protein’s functional groups and HA’s calcium ions [48]. Several factors, including the specific structural characteristics of the protein and HA’s surface properties, also influence the adsorption of BMP-2 to HA [48]. BMP-2 demonstrated a positive charge, resulting in the electrostatic attraction between HA and BMP-2 and further adsorption of BMP-2 on the HA surface [48]. The same studies also suggested the presence of hydrogen bonds between HA and BMP-2 based on FTIR experiments [48].

Additionally, extracellular Ca^2+^ ions have been reported to enhance BMP-2 effects on OCN, RUNX2, and osterix expressions in a calvaria critical-size defect model [49]. The interaction between extracellular Ca^2+^ and BMP-2 in osteoblasts was found to be induced by the calcium-dependent transcription factor NFATc1 of BMP-2 [49]. In the same study, exposure of Ca^2+^ ions to the cell culture also increased BMP-2, BMP-4, and Axin2 gene expression, confirming the positive relationship between calcium and BMP-2 [49]. Ca^2+^ and BMP-2 then cooperatively stimulate the osteogenic differentiation of the osteoblast [49]. 

The results suggest that the HA content in the CSMA-2 10HA scaffolds leads to increased BMP-2 expression and enhanced new bone formation compared to the CSMA-2 0HA group. This result might be attributed to the exposure of extracellular Ca^2+^ from the scaffold to the progenitor cells within the surrounding host tissue. This exposure appears to induce the BMP-2 secretion, resulting in a greater extent of new bone formation *in vivo*.

## 5. Conclusions

This study presents the result of *in vivo* experiments evaluating the biocompatibility of a 3D-printed CSMA-2 scaffold. The scaffolds were implanted in a subcutaneous model to evaluate the tissue response and in a rat calvaria critical-size defect model for the bone formation analysis. In the subcutaneous mode, the results demonstrated a favourable tissue response characterised by angiogenesis and fibrous tissue, indicating the early stage of the wound healing process. Meanwhile, the critical-size defect model showed new bone formation, as confirmed by micro-CT imaging and immunohistochemistry. The combination of CSMA-2 as the 3D printing material and gyroid as the 3D structure was found to support crucial events of bone healing, specifically angiogenesis and osteogenesis.

## Data Availability

Data are contained within the article.
